# *Campylobacter jejuni* Strain Dynamics in a Raccoon (*Procyon lotor*) Population in Southern Ontario, Canada: High Prevalence and Rapid Subtype Turnover

**DOI:** 10.3389/fvets.2020.00027

**Published:** 2020-02-11

**Authors:** Steven K. Mutschall, Benjamin M. Hetman, Kristin J. Bondo, Victor P. J. Gannon, Claire M. Jardine, Eduardo N. Taboada

**Affiliations:** ^1^National Centre for Animal Diseases, Canadian Food Inspection Agency, Lethbridge, AB, Canada; ^2^Department of Population Medicine, Ontario Veterinary College, University of Guelph, Guelph, ON, Canada; ^3^Department of Pathobiology, Ontario Veterinary College, University of Guelph, Guelph, ON, Canada; ^4^National Microbiology Laboratory at Lethbridge, Public Health Agency of Canada, Lethbridge, AB, Canada; ^5^Canadian Wildlife Health Cooperative, Ontario Veterinary College, University of Guelph, Guelph, ON, Canada; ^6^National Microbiology Laboratory, Public Health Agency of Canada, Winnipeg, MB, Canada

**Keywords:** *Campylobacter*, longitudinal surveillance, molecular subtyping, *Procyon lotor*, raccoon, zoonoses

## Abstract

Free-ranging wildlife are increasingly recognized as potential reservoirs of disease-causing *Campylobacter* species such as *C. jejuni* and *C. coli*. Raccoons (*Procyon lotor*), which live at the interface of rural, urban, and more natural environments, are ideal subjects for exploring the potential role that wildlife play in the epidemiology of campylobacteriosis. We studied the prevalence and genetic diversity of *Campylobacter* from live-captured raccoons on five swine farms and five conservation areas in southwest Ontario. From 2011 to 2013, we collected fecal swabs (*n* = 1,096) from raccoons, and (*n* = 50) manure pit samples from the swine farm environment. We subtyped the resulting *Campylobacter* isolates (*n* = 581) using Comparative Genomic Fingerprinting (CGF) and 114 distinct subtypes were observed, including 96 and 18 subtypes among raccoon and manure pit isolates, respectively. *Campylobacter* prevalence in raccoons was 46.3%, with 98.7% of isolates recovered identified as *C. jejuni*. Novel raccoon-specific CGF subtypes (*n* = 40/96) accounted for 24.6% (*n* = 143/581) of *Campylobacter* isolates collected in this study. Our results also show that *C. jejuni* is readily acquired and lost in this wild raccoon population and that a high *Campylobacter* prevalence is observed despite transient carriage typically lasting 30 days or fewer. Moreover, although raccoons appeared to be colonized by species-adapted subtypes, they also harbored agriculture-associated genotypes that accounted for the majority of isolates observed (66.4%) and that are strongly associated with human infections. This suggests that raccoons may act as vectors in the transmission of clinically-relevant *C. jejuni* subtypes at the interface of rural, urban, and more natural environments.

## Introduction

*Campylobacter* species are the second most reported bacterial foodborne pathogen in Canada (1), and campylobacteriosis remains one of the most common enteric illnesses worldwide (2). Although human infections are primarily caused by two thermophilic species, *C. jejuni* and *C. coli*, several additional species have been reported to cause illness (3). It is generally accepted that the majority of *Campylobacter* infections are acquired through the handling and ingestion of contaminated poultry products (4–6). While *Campylobacter* is highly prevalent in poultry and poultry meat (7), it is also commonly found in a wide range of animal hosts that not only include livestock such as cattle and swine, but also household pets and wildlife (6, 8). Given that *Campylobacter* is actively shed in animal feces, environmental sources such as surface waters may also become contaminated through use by animals or due to surface run-off (9). This may lead to increased environmental transmission and dissemination of *Campylobacter* across different ecological niches, thereby creating additional routes of exposure to humans or other potential hosts (10).

*Campylobacter* have been isolated from a diverse array of free-ranging wildlife, including birds such as waterfowl and songbirds and mammals, including rodents, wild boars, and ungulates (8, 11–14). Although the presence of *C. jejuni* in free-ranging wildlife is not necessarily indicative of a role in the epidemiology of campylobacteriosis, an increasing number of studies is consistent with this possibility. For example, *Campylobacter* subtypes commonly associated with human illness have been detected in wildlife species (15, 16), and human clinical cases have been linked to wildlife via direct transmission (17–20) or source attribution (21). Furthermore, an increased occurrence of zoonotic pathogens, including *Campylobacter* and *Salmonella*, has been observed in free-ranging wildlife living in close proximity to livestock and or human populations (22, 23).

A recent scoping review on bacterial zoonotic pathogens in wild animals reported that the most frequently investigated wildlife groups were birds (47.3%), cervids (15.4%), and rodents (10.5%) (24). Similarly, much *Campylobacter* research on wildlife has focused on avian species, in large part due to *Campylobacter*'s affinity for avian hosts, but also due to host ecological factors such as increased anthropogenic contact and large habitat ranges. The importance of wild mammals in *Campylobacter* ecology and epidemiology is less clear. By comparison to avian species, there is a paucity of literature exploring the role of non-avian wildlife on transmission of *Campylobacter*. There is even less focus on synanthropic species, which live in habitats in close association with humans. In this regard, raccoons (*Procyon lotor*) represent ideal study subjects because they use a wide variety of habitats (25) and have the potential to move between urban, rural, and forested habitats.

Raccoons are known to carry a number of zoonotic agents (26–29) and they also display distinct social features such as the use of communal latrines (30), which may enhance mechanical transmission of certain microorganisms present in their feces (31). Previous studies have reported *Campylobacter* prevalence in raccoons ranging from 1% (32) to 41% (33). To our knowledge, there have been few, if any, longitudinal studies examining *Campylobacter* in a raccoon population over multiple years. The objectives of this study were to: (1) determine the prevalence of *Campylobacter* in raccoons captured on swine farms and conservation areas; (2) assess the genetic diversity, population structure, and ecology of *Campylobacter* subtypes observed in raccoons; (3) assess the dynamics of *Campylobacter* acquisition/loss in individual animals; and finally, (4) compare the subtypes observed in raccoons and human clinical cases to assess the potential role of raccoons in the epidemiology of campylobacteriosis.

## Materials and Methods

### Animal Trapping and Sample Collection

Procedures for trapping and handling raccoons were approved by the Animal Care Committee at the University of Guelph following the guidelines of the Canadian Committee on Animal Care. For a detailed description and map of the study area as well as trapping and sampling procedures, please refer to Bondo et al. (27) and (29). From May 2011 to November 2013, raccoons were live-trapped on swine farms and conservation areas near the cities of Guelph and Cambridge in southern Ontario, Canada. Individual animals were identified by ear and transponder tags and were sampled only once per monthly trapping week; however, additional samples were collected from the same individual if they were caught in subsequent months. Rectal fecal swabs were collected using Cary-Blair swab applicators [BBL CultureSwab, (BD) Becton, Dickinson and Company, Annapolis, MD, USA]. At each swine farm, one lagoon (i.e., manure pit) sample was collected on the first day of each trapping week as previously described (29). Briefly, to collect lagoon samples, a 24′ Nasco Swing Sampler (Conbar, Monroeville, NJ, USA) was used to collect three sub-samples from three locations around the pit, and up to two depths (i.e., the top 1/3, and mid depth of the storage), for a total of six sub-samples. The samples were then pooled into one sample for analysis. All samples were kept refrigerated or on ice until processing. The median and average number of days between collection and processing was three; the maximum number of days for raccoon swabs was eight whereas for manure pit samples it was 11.

### *Campylobacter* Isolation

Isolation of thermophilic *Campylobacter* species was performed as previously described (34). For samples collected prior to October 2011, the “conventional method” (enrichment followed by direct plating onto selective media) was used; the “membrane method” (enrichment followed by passive membrane filtration onto selective media) was used thereafter. Briefly, fecal swabs were immersed in 20 mL of Bolton broth (BB) containing BB selective supplement (SR0183, Oxoid), (20 mg/L cefoperazone, 20 mg/L vancomycin, 20 mg/L trimethoprim, and 50 mg/L cyclohexamine) and 5% laked horse blood (SR0048) and mixed rapidly for 20 s. Lagoon samples were mixed prior to adding 1 mL of sample to BB with supplement. Bolton broth cultures were incubated at 42°C in microaerobic conditions (10% CO_2_, 5% O_2_, 85% N_2_) for 24 h and subsequently streaked onto modified blood-free charcoal cefoperazone deoxycholate agar supplemented with 32 mg/L cefoperazone and 10 mg/L amphotericin B (mCCDA) or passively filtered for 15 min through 0.65 μM cellulose acetate membrane filters onto the surface of mCCDA. All mCCDA plates were incubated for 48 h under the same microaerobic conditions. Three to five *Campylobacter*-like colonies were subcultured to blood agar and incubated for 24–48 h. Presumptive *Campylobacter* spp. colonies were identified on the basis of growth on mCCDA media, colony morphology, and oxidase tests. DNA was extracted from purified cultures using the EZ1 DNA tissue kit (Qiagen) according to the manufacturer's instructions for subsequent PCR speciation of presumptive *Campylobacter* isolates and subtyping of confirmed *Campylobacter* isolates.

### PCR Confirmation of *Campylobacter* spp.

Presumptive *Campylobacter* isolates were confirmed by multiplex PCR targeting a *Campylobacter* genus-specific region of the 16S *rRNA* gene, and *mapA* and *ceuE* genes for *C. jejuni* and *C. coli* identification, respectively (35). Amplicons were visualized using a QIAxcel capillary electrophoresis instrument with the DNA Screening kit. The AM320 separation method was used along with a 15–3,000 bp alignment marker and the QX 100–2.5 kb DNA size marker. Data were analyzed and visualized using the BioCalculator v. 3.0 software.

### Comparative Genomic Fingerprinting

*Campylobacter* spp. isolates were subtyped by Comparative Genomic Fingerprinting (CGF) as previously described (36). Briefly, CGF consists of 8 multiplex PCR reactions that together assess the presence or absence of a set of 40 accessory gene targets found to have variable carriage in the *Campylobacter* population and that are used to generate a highly discriminatory binary fingerprint. Products from the CGF PCRs were visualized on the QIAxcel as previously described (36) and were scored positive (1) or negative (0) based on presence or absence of each target amplicon using a combination of the BioCalculator software's binary peak calling and confirmed with visual curation. The resulting binary fingerprints were assigned a three-digit CGF subtype (e.g., 0923.002.001) derived from cluster membership in the Canadian *Campylobacter* CGF database (C3GFdb). Fingerprints identical to those already existing in the database were assigned the appropriate CGF subtype, while novel fingerprints were assigned a CGF subtype based on their similarity to existing fingerprints in the database. Isolates from the same sample with identical subtypes were assumed to be derived from a single clone and only one representative isolate was included in further analyses.

### Association of CGF Type With Host Species

At the time of this analysis, the C3GFdb consisted of 4,847 distinct CGF profiles obtained from 19,141 *Campylobacter* isolates collected from across Canada, primarily from the last decade. These included 23.3% isolates derived from human clinical origin, 32.0% from poultry sources, 20.9% from cattle sources and 14.6% from environmental surface water samples. To compare epidemiologic attributes of subtypes observed among study isolates, we compiled summary statistics based on the composition of host-sources observed in the C3GFdb for each subtype in this study. We classified CGF subtypes observed in this study (*n* = 114) into four ecological range categories (ERC) based on the composition of sources from which they have been historically observed in the C3GFdb. Subtypes from the present study included: (I) Raccoon Exclusive (RE; *n* = 44); (II) Raccoon and Environmental Water (REW; *n* = 16); (III) Swine/Swine Lagoon (SSL; *n* = 10); and (IV) Mixed Host (MH; *n* = 44). Phylogenetic and source association analysis was performed using the R language for statistical computing (v.3.13) (37) and the following packages: *tidyverse* (38) and *ggtree* (39). A dendrogram was constructed from the 40-gene CGF profile using the “hamming” distance and “average linkage” clustering from the *dist* and *hclust* functions, respectively, and visualized using “ggtree.” Clade designations were made based on a tree height “*h* = *10*,” which produced stable, genetically distinct groupings. For each of the CGF subtypes observed in the present study we identified the associated host-sources from the C3GFdb. For associations between CGF subtypes and MLST Clonal Complexes, we used an in-house database created from *in silico* CGF and MLST predictions generated from publicly available WGS data analyzed using the program Microbial *in silico* Typer (40); a table of associations is provided as Supplementary Table S1.

### Analysis of Strain Dynamics

Forty-three percent (*n* = 272/628) of the raccoons in this study were captured on multiple occasions, with 18.5% (*n* = 116/628) captured three or more times, up to maximum of eight captures. These data were analyzed to examine strain dynamics at the level of individual animals as described below.

### Statistical Analysis

CGF subtype diversity was assessed using the Simpson's Diversity Index (41) with a 95% confidence interval (42). A chi-square test statistic was used to explore the hypothesis that there was no significant difference in the distribution of isolates from prevalent *Campylobacter* clades (isolate count n > 9) between each of the location types (e.g., swine farm sites and conservation sites). Chi-square test statistics and *post-hoc* follow-up calculations for computing adjusted standardized residuals and z-scores for each clade were performed as described by Sharpe (43).

## Results

### A High Prevalence of *Campylobacter* Was Found in the Raccoon Population Under Study

From May 2011 to November 2013, we collected 1,096 fecal swabs from 628 raccoons trapped on conservation areas (*n* = 687) and swine farms (*n* = 409), and 50 manure pit samples from the environment of the swine farm sites. The prevalence of *Campylobacter* spp. in raccoon fecal samples was 46.3% (508/1,096) (Table 1). There were no significant differences in *Campylobacter* prevalence from raccoon fecal swabs with a sample-to-test interval of 1–2 days (46.7%, *n* = 467) vs. a 3–4 days (46.0%, *n* = 363), 5–6 days (48.5%, *n* = 227), or 7–8 days interval (33.3%, *n* = 39). Similarly, for swine manure pit samples, there were no significant differences in Campylobacter prevalence between sample-to-test intervals of 1–2 days (40.0%, *n* = 10) vs. 3–4 (38.1%, *n* = 21), 5–6 (33.3%, *n* = 9), or 7–11 days (22.2%, *n* = 9). Among the *Campylobacter* positive raccoon samples, 502 (98.8%) were positive for *C. jejuni*, six (1.2%) for *Campylobacter* spp. (unidentified *Campylobacter* species), and one for *C. coli* (Table 1). A single sample was found to harbor mixed *Campylobacter* species, testing positive for both *C. jejuni* and an undefined *Campylobacter* spp. Among swine manure pit samples 34.0% (17/50) were positive for *Campylobacter*, of which 94.1% (16/17) were positive for *C. coli* (Table 1). One sample tested positive for *Campylobacter* spp.

**Table 1:** Frequency, prevalence and species of *Campylobacter* in fecal samples obtained from raccoons and swine farm manure pits.

**Sample source**	**Sample size**	***Campylobacter*** **positive samples**		**(95% confidence interval)**	***C. jejuni***		***C. coli***		***C***. **spp**.	
	***n***	***n***	**%**		***n***	**%**	***n***	**%**	***n***	**%**
Raccoon	1,096	508	46.4	(0.434, 0.493)	502	98.8	1	0.2	6	1.2
Manure pit	50	17	34.0	(0.224, 0.478)	0	0.0	16	94.1	1	5.9

### *Campylobacter* Circulating in the Raccoon Population Represent a Genetically Diverse Population

**Figure 1:**
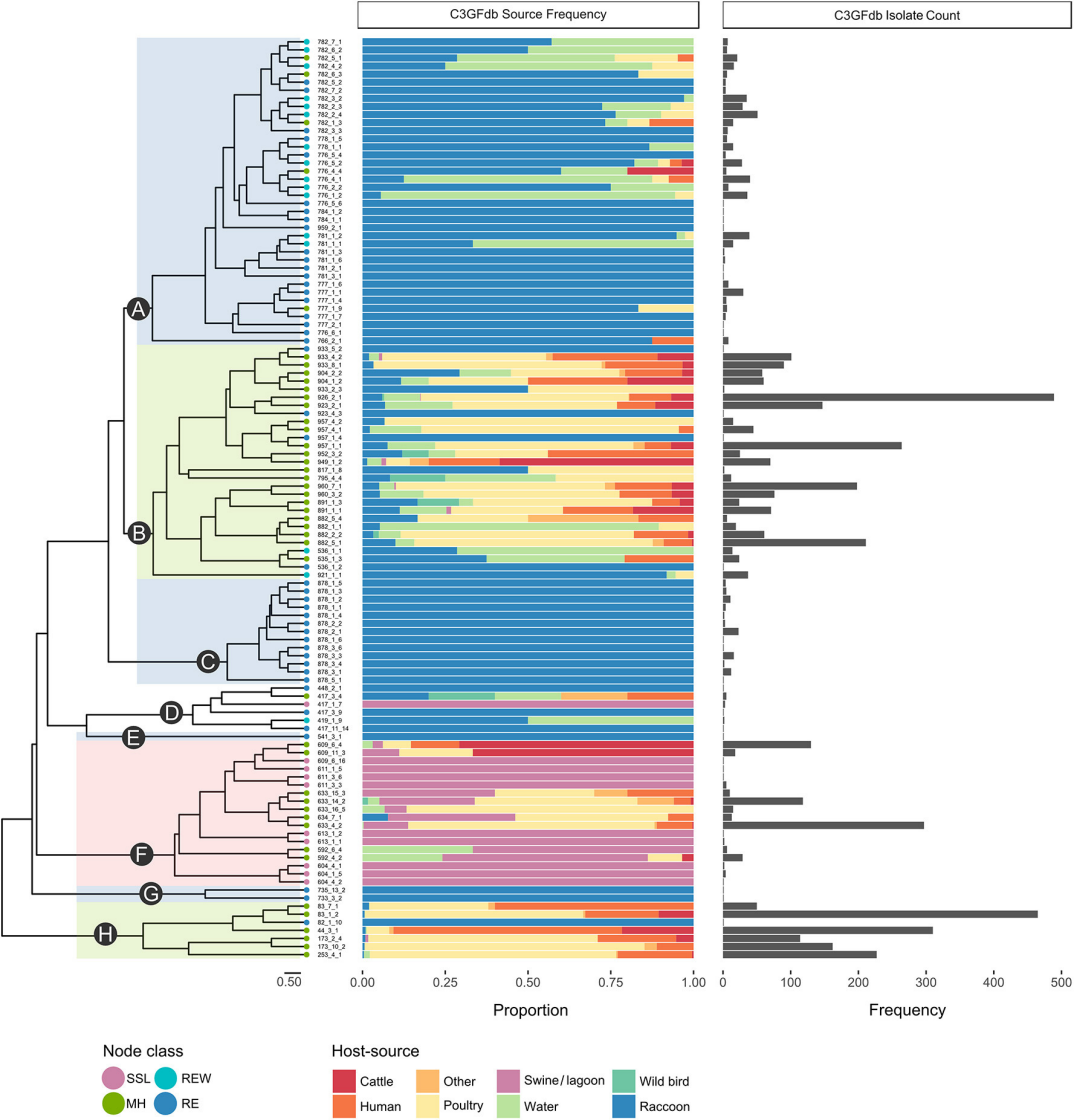
Host-range specificity of *Campylobacter* subtypes observed in a raccoon population on conservation and swine farm sites in southwest Ontario, Canada. For each of the 114 CGF types in the dendrogram, host-source data from the C3GFdb is used to illustrate relative host-source associations. Terminal nodes of the tree were classified based on source composition of the CGF subtype (MH, mixed host; RE, raccoon exclusive; REW, raccoon and environmental water; SSL, swine and swine lagoon). A large proportion (~97%) of Raccoon isolates from this study are found in clades A, B, and C. Both clade A, which is largely composed of RE and REW subtypes, and clade C, which consists entirely of RE subtypes, appear to be raccoon-associated niche or host-adapted lineages. Clade B is primarily composed of MH subtypes with a large contribution from agriculture and human clinical sources.

A total of 1,555 confirmed *Campylobacter* isolates derived from *Campylobacter*-positive raccoon fecal swabs were subtyped by CGF. A further 58 isolates were recovered from swine manure pit samples. After accounting for potentially clonal isolates (i.e., isolates from the same sample sharing the same CGF subtype), 610 isolates remained; this included 581 raccoon isolates and 29 manure pit sample isolates. Among the *Campylobacter* positive raccoon samples (*n* = 508), a single CGF subtype was recovered for the majority of the samples (443/508; 87.2%), two CGF types were recovered in 11.2% of samples (57/508), and three subtypes in 1.6% of samples (8/508). We observed a broad genetic diversity in the *Campylobacter* population, with a total of 114 distinct CGF subtypes identified among study isolates. Subtype clusters ranged in size from 1 to 36 isolates, with 13 subtypes comprising over 50% of isolates in the dataset (*n* = 329/628) and 51 subtypes detected in single instances. Forty-six subtypes, representing 21.3% of study isolates (*n* = 130/610), were novel and had not been previously observed in the C3GFdb. There were 96 subtypes observed among raccoon isolates (*C. jejuni* = 91; *Campylobacter* spp. = 4; *C. coli* = 1) and 18 subtypes observed among lagoon isolates (*C. coli* = 17; *Campylobacter* spp. = 1); no subtypes were shared between raccoons and manure pit samples. Clustering of the 114 CGF binary profiles revealed 8 major lineages, designated clades A–H (Figure 1). The clades segregated by *Campylobacter* species: clades A–C, G, and H comprised *C. jejuni* isolates; clade F comprised *C. coli* isolates; and clade D comprised non-*jejuni*/*coli Campylobacter* species isolates (i.e., undetermined). The majority of raccoon isolates were observed in clade A (52.0%; *n* = 302), followed by clades B and C, with 30.5% (*n* = 177) and 14.5% (*n* = 84) of isolates, respectively. A small number of raccoon isolates (3.1%; *n* = 18) were found distributed among clades D (*n* = 5), E (*n* = 1), F (*n* = 1), G (*n* = 2), and H (*n* = 9). Among swine lagoon isolates, the majority were found in clade F (89.7%; *n* = 26), with the remaining isolates (10.3%; *n* = 3) in clade D. Similar levels of diversity were observed between swine farms and conservation areas at the subtype level (Simpson's Index of 0.9648 and 0.9628, respectively). Although overall genotypic richness was similar between swine farms and conservation areas, there was a significant relationship between clade and location type. For clade A, more isolates were identified from swine farm sites compared to conservation sites (*p* < 0.001). In contrast, for clades B and C, fewer isolates were identified from swine farm sites compared to conservation sites (*p* < 0.01).

### *Campylobacter* Subtypes in the Raccoon Population Display Different Types of Host Range Specificity

Among the four ecological range categories that we defined for the subtypes in this study (Raccoon Exclusive or RE; Raccoon and Environmental Water or REW; Swine/Swine Lagoon or SSL; and Mixed Host or MH), raccoon isolates were evenly distributed among RE, REW and MH subtypes (RE: 31.3%, *n* = 182; REW: 34.1%, *n* = 198; MH: 34.6%, *n* = 201). Swine lagoon isolates were similarly distributed among SSL and MH subtypes (SSL: 51.7%, *n* = 15; MH: 48.3%, *n* = 14). The distribution of ERC subtypes suggests a strong raccoon association for certain clades observed in our analysis (Figure 1). Clade C, which included 14.5% of raccoon isolates in the study, was strictly composed of RE subtypes, and these were novel to the C3GFdb. Clade A, which included the majority of raccoon isolates in the study (52.0%), comprised largely RE and REW subtypes (*n* = 33/38). Source frequencies from the C3GFdb (Table 2) indicate that a majority of isolates from clade A subtypes (*n* = 461) include raccoon isolates from this study (65.5%; *n* = 302) along with historical isolates from environmental water sources (26.2%; *n* = 121). A small number of isolates (6.9%; *n* = 32) comprised the remaining isolates in the clade and were derived from cattle, poultry, and human clinical sources (Table 2). Conversely, clades B and H, which included 30.5 and 1.6% of raccoon isolates, respectively, were of MH subtype composition (*n* = 23/29 and *n* = 6/7, respectively). Based on C3GFdb source association data, isolates from subtypes in clades B (*n* = 2,118) and H (*n* = 1,329) were primarily chicken-associated (clade B = 54.9%; clade H = 55.0%). Importantly, these clades included a large proportion of isolates from human clinical cases (14.4 and 33.3%, respectively), which is in contrast to clades A and C (1.7 and 0.0%, respectively). Unsurprisingly, the *C. coli* specific-clade F included 26/29 swine manure pit isolates and consisted of 9 SSL subtypes and 9 MH subtypes.

**Table 2:** Host-source distributions from the C3CGFb for each of the clades observed in raccoon and swine manure pit isolates.

**Clade**	**CGF subtypes**	**C3CGFdb source distribution**								**This study**		**Total isolates**
		**Human clinical**	**Cattle**	**Poultry**	**Other**	**Swine**	**Wild bird**	**Wild mammals**	**Water**	**Raccoon**	**Manure pit**	
A	38	8	2	22	0	0	0	6	121	302	0	461
B	29	306	171	1,163	33	5	11	15	237	177	0	2,118
C	13	0	0	0	0	0	0	1	0	84	0	85
D	6	1	0	0	1	0	1	0	2	5	3	13
E	1	0	0	0	0	0	0	0	0	1	0	1
F	18	60	107	319	16	104	2	0	19	1	26	654
G	2	0	0	0	0	0	0	0	0	2	0	2
H	7	443	123	731	15	1	0	2	5	9	0	1,329

*Clade C was exclusively composed of raccoon isolates, while clade A was largely of raccoon and environmental water origin. Clades B and H showed broad source distributions and have been associated with “generalist” lineages. Clade G, a C. coli group, also showed a broad source distribution and included all C. coli swine lagoon isolates*.

### *Campylobacter* Strain Dynamics in Raccoons Include Both Transient and Longer-Term Carriage Within Individual Animals

**Figure 2:**
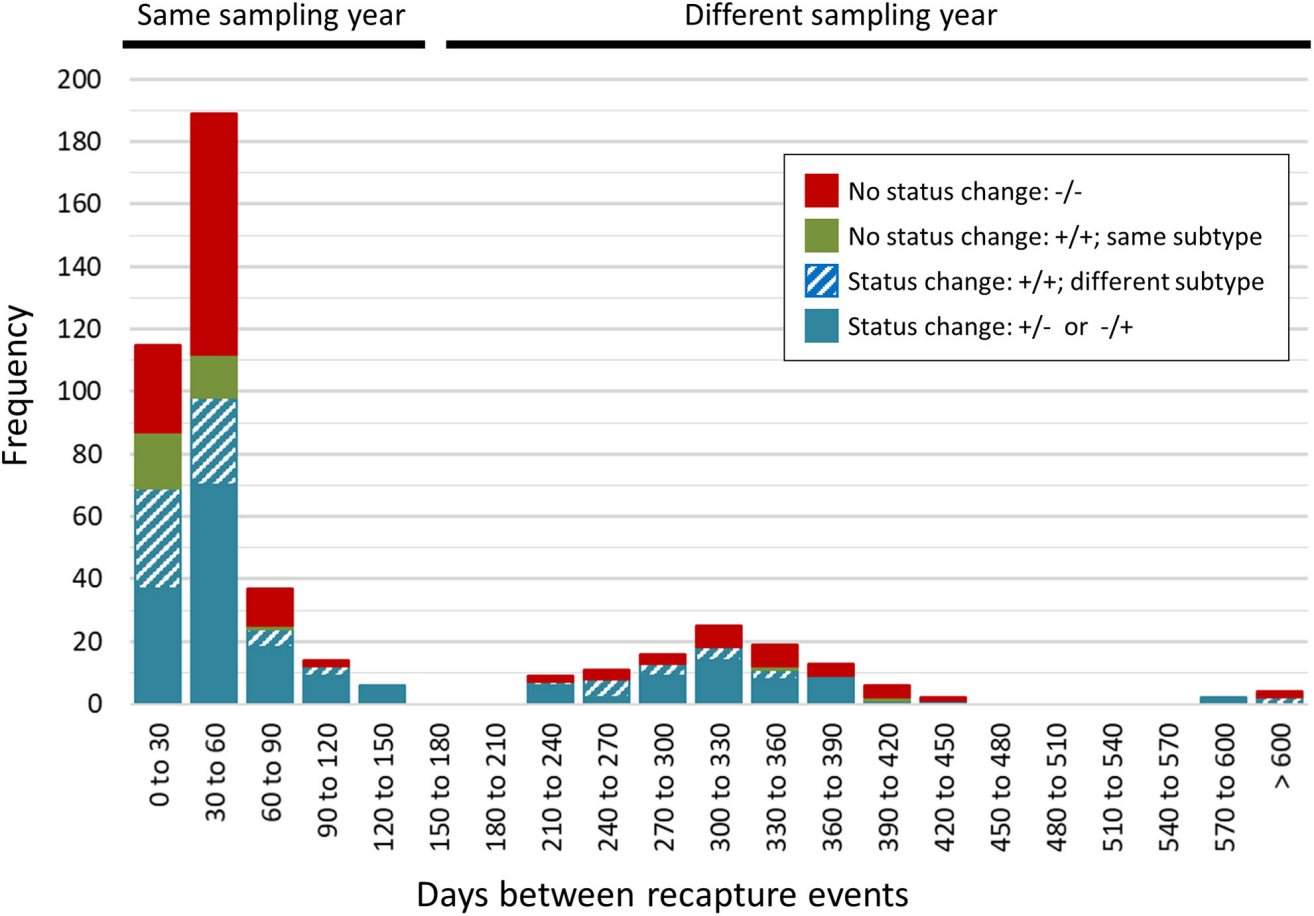
Rapid changes in *Campylobacter* status in a raccoon population on conservation and swine farm sites in southwest Ontario, Canada. *Campylobacter* culture status was examined for consecutive recapture events involving the same animal (*n* = 468). A majority of consecutive recapture events (77%; *n* = 361) occurred within the same sampling year and 67% (*n* = 242/361) yielded a *Campylobacter* positive result. The positive rate among recapture events in different sampling years was 69% (*n* = 74/107). Overall, 89% of recapture events that involved a *Campylobacter* positive result (*n* = 281/316) reflected a *Campylobacter* status change, with either animals that tested positive after previously testing negative or vice-versa (*n* = 191), and animals testing positive in both cases but that shed a different subtype (*n* = 90). These strain dynamics are consistent with the rapid turnover of strains in this raccoon population.

**Figure 3:**
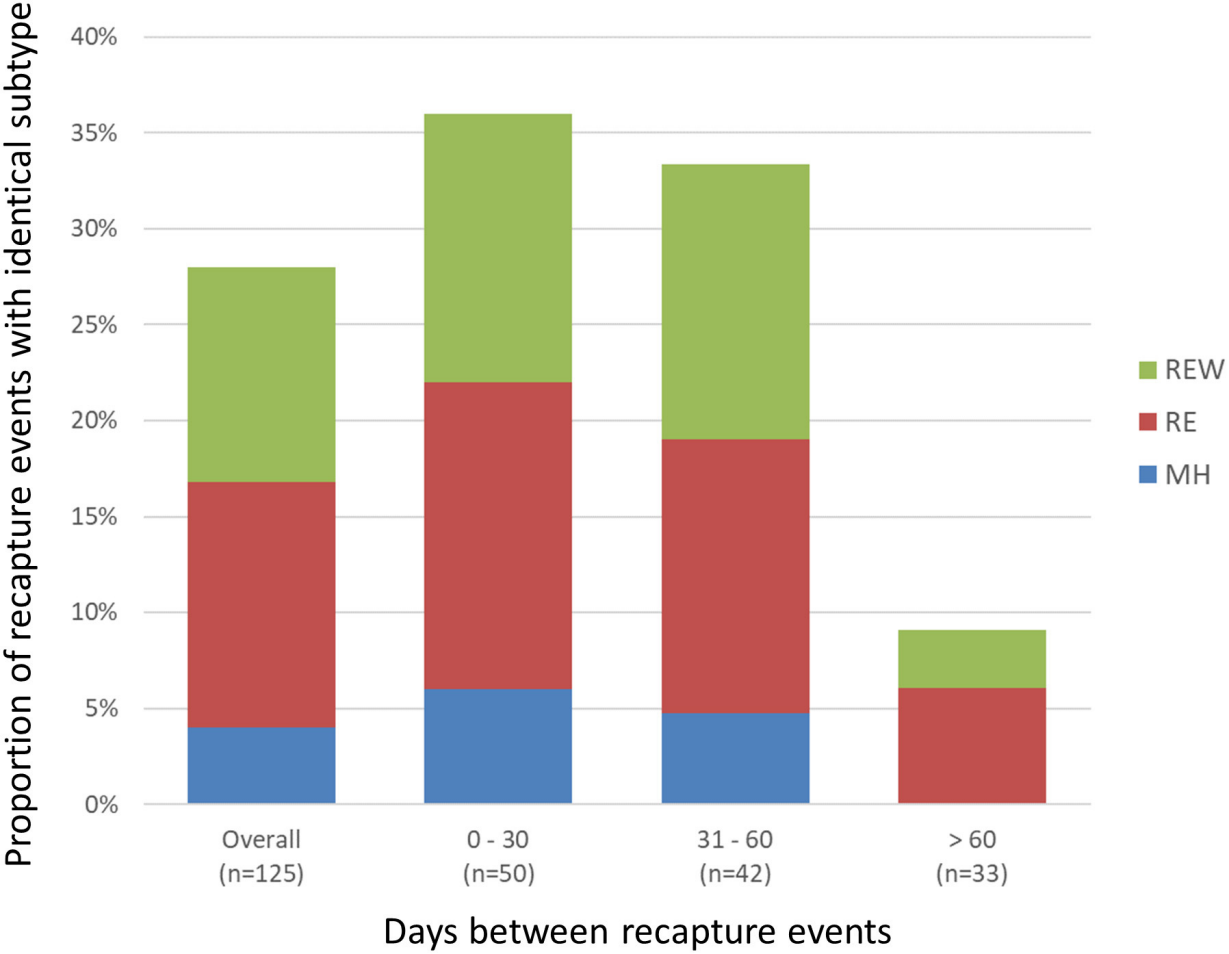
Short- vs. long-term colonization with *Campylobacter* in a raccoon population on conservation and swine farm sites in southwest Ontario, Canada. Analysis of consecutive *Campylobacter* positive recapture events yielding an identical subtype indicates that as the duration between captures increased beyond 60 days, there is a large decrease in the number of cases where the same subtype was observed, suggesting that most animals are only transiently colonized. Among the small proportion of recapture events consistent with prolonged colonization (i.e., consecutive and temporally separated *Campylobacter-*positive recapture events yielding the same subtype), results indicate that these are more likely to involve raccoon-adapted subtypes from RE and REW categories.

A total of 468 samplings (43%; *n* = 468/1,096) involved consecutive recapture events involving the same animal (Figure 2), with a majority (77%; *n* = 361/468) occurring within the same sampling year. The positive rate for *Campylobacter* was 67% (*n* = 242/361) and 69% (*n* = 74/107) among recaptures in the same and different sampling years, respectively (Figure 2). Among recaptures within the same sampling year, 58% (*n* = 209/361) reflected a *Campylobacter* status change, with either animals that acquired or lost *Campylobacter* (*n* = 139), and animals that acquired a different subtype (*n* = 70) between recaptures. This rate was 67% among recapture events in different sampling years (*n* = 72/107), including 52 cases of animals acquiring or losing *Campylobacter* between recaptures and 20 cases of animals acquiring a different subtype. Overall, 60% (*n* = 281/468) of recapture events reflected a change in *Campylobacter* status, although the rate rises to 89% (*n* = 281/316) when excluding recaptures in which the animal tested negative on both occasions (*n* = 152). There were 125 instances of consecutive positive results, with a median of 35 days between recaptures (minimum = 25 days, maximum = 617 days). Of these, 40.0% (*n* = 50/125) occurred within a window of 0–30 days (i.e., short), an additional 33.6% (*n* = 42/125) occurred in a window of 31–60 days (i.e., intermediate), and 26.4% (*n* = 33/125) occurred in a window of >60 days (i.e., long), which included 22 recaptures that occurred on different sampling years. Overall, 72.0% of consecutive recaptures that were positive on both occasions (*n* = 90/125) yielded a different subtype on consecutive samplings. Among the 28% of consecutive recaptures yielding the same subtype (*n* = 35/125), we observed a decreasing proportion of matching subtypes as the time between sampling events increased (Figure 3). Furthermore, when examined in the context of ecological range, we observed that a larger proportion of recaptures in which the same subtype was consecutively isolated involved raccoon-associated subtypes (i.e., categories RE and REW) compared to MH subtypes (Overall: 30/35; 0–30 days: 15/18; 31–60 days: 12/14; and >60 days: 3/3). Among recapture events yielding changes in *Campylobacter* status on consecutive observations, the ratio of instances of strain acquisition to strain loss was similar across the three major raccoon-containing clades (A: 126/90; B: 66/46; C: 41/24 and across the various ERCs (MH: 73/57; RE: 80/53; REW: 87/56), all of which were similar to the overall ratio of acquisition-to-loss (*n* = 240/166).

## Discussion

The prevalence of *Campylobacter* spp. in raccoon fecal samples in this study was 46%, which is consistent with what was observed in a previous study from the same region of southwestern Ontario (41%) (33). Indeed, given the practical limitations imposed by potential delays in sample processing (3–11 days) due to shipping of fecal swabs from the study site (Guelph, Ontario) to the laboratory where microbiological work was carried out (Lethbridge, Alberta), it is very likely that our prevalence estimates underestimate true prevalence rates in this raccoon population. By contrast, studies from Japan and New York found only 1.3 and 6% of raccoons carried *Campylobacter*, respectively (32, 44). There are a limited number of studies systematically examining *Campylobacter* in raccoons or other medium sized mammals. Many studies instead rely on convenience-based sampling methodology, for example, intakes into a wildlife rehabilitation center (20, 45). The Grand River watershed in southern Ontario is heavily impacted by mixed agricultural activities, with farms making up ~75% of the watershed (46).

In this study, we examined raccoons circulating in a range that included several swine farms and more natural environments in order to investigate *Campylobacter* prevalence, genetic diversity, and strain dynamics in this wild animal population. Significant genetic diversity was observed among raccoon isolates collected in this study, with 96 distinct CGF subtypes and at least eight major lineages observed in this population. Interestingly, there was a distinct lack of overlap in CGF subtypes observed in raccoons trapped at swine farm sites and samples obtained at those farms (i.e., manure pit samples) despite the high rates of *Campylobacter* recovery in these animals. Swine are known to preferentially harbor *C. coli*, which was recovered from nearly all manure pit samples analyzed. The near absence of *C. coli* in raccoons from this study was unexpected, as some level of *C. coli* exposure would be expected to take place in the swine-farm environment. Nonetheless, our findings are consistent with overall trends in the C3GFdb, in that of nearly 300 subtypes that have been observed in either raccoons or swine, only seven minor subtypes have included isolates recovered from both species. Other studies investigating interactions between wildlife and swine farms have shown little evidence of shared *Campylobacter* subtypes between farm fecal/manure isolates and small and medium sized wild mammals (such as mice, rats, badger, fox) (11, 47, 48). The swine in this study were housed indoors, which likely prevented raccoons from coming into direct contact with the animals or fresh swine feces. Furthermore, swine fecal wastes are generally managed in a more confined manner (e.g., manure pits) compared to other livestock such as cattle. Lastly, because raccoons have wide-ranging habitats, it is possible that raccoons trapped at swine farm sites may not have routinely used these areas to forage, thereby decreasing their exposure. Thus, there exist possible barriers (i.e., biological, physical, behavioral) that may limit the transmission of *C. coli* between swine and raccoons.

During the course of this longitudinal study, a significant proportion of samples represented cases in which the same animal was recaptured and these data were used to examine *Campylobacter* strain dynamics within individual animals, including patterns of short-, intermediate-, and long-term *Campylobacter* carriage and their shedding. A majority of recapture events (60%) yielded differences in *Campylobacter*-status, including cases in which the animal appeared to have acquired or lost *Campylobacter* between recaptures (40%), and cases in which the animal tested positive in both occasions but where we observed a difference in subtype (19%). Overall shifts in *Campylobacter* status increased to 89% if excluding recaptures in which the animal tested negative in both occasions. Moreover, we observed instances of *Campylobacter*-positive animals that appeared to revert to negative status on subsequent recaptures in as few as 25 days, with 18 instances occurring within 30 days. This would suggest that although *Campylobacter* prevalence in this raccoon population is high, carriage and shedding is likely transient, with the majority of raccoons harboring *C. jejuni* for only short periods of time (≤1 month). We also observed significant strain displacement among animals that tested positive for *Campylobacter* on consecutive recaptures, with a different subtype observed in 72% of cases. Moreover, although we selected 1,555 isolates for subtyping from among the 508 fecal swabs that tested positive for *Campylobacter*, a majority of samples (87.2%) yielded a single subtype. Taken together, these data suggest that raccoons readily acquire and lose *Campylobacter*, with rapid turnover of strains among animals that remained *Campylobacter*-positive on consecutive samplings. Nonetheless, it is important to note possible confounding factors that could yield data consistent with transient carriage. These include limitations with microbiological and molecular subtyping methods (i.e., limit of detection/recovery of isolates leading to culture-negative samples or limiting the isolation of multiple strains from a single sample).

Our data yielded a small proportion of instances in which consecutive recaptures were *Campylobacter* positive and yielded the same subtype. Although these tended to be recapture events on consecutive months, a small number of cases included recaptures with significant temporal separation, up to a length of 394 days. It is unclear whether these represent ongoing colonization or re-infection with the same subtype; whole-genome sequence (WGS) analysis of these isolates is likely to shed light on this issue since the much higher discriminatory power of WGS-based subtyping approaches could help differentiate between these two types of events.

Our analysis of host source metadata from the Canadian *Campylobacter* CGF database (C3GFdb) revealed that the *C. jejuni* population found in raccoons consisted primarily of subtypes with a strong raccoon association and subtypes with a mixed-host association. These were primarily distributed among three major clades that included the majority of raccoon isolates in this study. Nearly one-third of raccoon isolates had subtypes that were novel to the C3GFdb, with many of these forming a clade that appears to be exclusive to raccoons. Most remaining isolates from raccoon-exclusive subtypes could be found in a clade that was otherwise composed primarily of subtypes that had previously been observed only among isolates from surface water samples. Host-adapted subtypes in wild animals and surface water have been described in previous studies (49–51). Although Stabler et al. (8) have previously described an apparent water and wildlife *C. jejuni* clade, this is the first report of *Campylobacter* subtypes that appear to be uniquely host-adapted to raccoons. It is noteworthy that the majority of events in which animals were positive for the same CGF subtype on consecutive recaptures (i.e., consistent with longer-term colonization) involved raccoon-adapted subtypes.

In contrast to raccoon-adapted clades, 78% of isolates in clade B, which contained approximately 30% of raccoon isolates in this study, were from subtypes with mixed-host association. Mixed-source genotypes have been well-described and typically include representatives from Clonal Complexes (CCs) described as ‘generalists' including ST-45, ST-21, and ST-48 (52–55). These CCs are highly prevalent worldwide, known for their broad host distribution, including livestock, and high burden of human illness. During the course of validating CGF (36), we established correlations between specific CGF subtypes and corresponding CCs and have subsequently been able to refine these based on *in silico* subtyping predictions from WGS data (40, 56). Notably, many CGF subtypes within clade B have been characterized as belonging to CC ST-45, including CGF subtype 0926.002.001, which is the third most prevalent genotype in the C3GFdb and ranks sixth in terms of number of human clinical cases associated with it. Although clade H only comprised a small proportion of raccoon isolates in this study (1.6%), it is noteworthy because CGF subtypes from within clade H have been shown to belong to CCs ST-21 and ST-48 (57). Moreover, it includes CGF subtypes 0044.003.001 and 0083.001.002, which are among the most prevalent in Canada and rank second and third in terms of number of associated human clinical cases, respectively. Because of their synanthropic behavior, the potential for acquisition of clinically relevant *Campylobacter* subtypes by raccoons via the same routes of exposure as humans cannot be ruled out. Our data suggest that the high *Campylobacter* rates observed in this wild raccoon population are likely due to environmental and ecological factors, including high rates of mixed agriculture activities, which allow for sustained and consistent *Campylobacter* exposure to agriculture-associated genotypes strongly implicated in human cases of campylobacteriosis.

## Conclusions

Prevalence of *Campylobacter* in the raccoon population under study, which is native to the Grand River Watershed in southwestern Ontario, Canada, was found to be much higher than what has been previously reported for other populations of small to medium-sized wild mammals. This high prevalence was observed despite evidence suggesting that individual animals were only transiently colonized, with a majority of raccoons harboring *C. jejuni* for only short periods of time (i.e., ≤1 month). Our data show significant genotypic flux within individual animals, which is consistent with the constant acquisition, loss, and replacement of strains, and suggest that this raccoon population is constantly exposed to a wide range of circulating but endemic strains. This may be due to ecological factors within the Grand River Watershed, where agriculture and human activities may give rise to a wide variety of *Campylobacter* sources. Although raccoons appear to be poor hosts for *C. coli* strains typically observed in swine, many of the *C. jejuni* subtypes observed in this raccoon population have been previously associated with agricultural sources (e.g., chickens, cattle) and human illness. Interestingly, raccoons were also found to carry *C. jejuni* subtypes within two clades that appear to be genetically distinct from genotypes recovered from humans and food animals and that appear to represent raccoon-associated niche- or host-adapted strains; the potential risk to human health from these strains remains unknown. Nevertheless, the high proportion of clinically-relevant generalist *Campylobacter* subtypes found in raccoon fecal samples suggests that raccoons likely act as transient vectors of *Campylobacter* and may play a role in the transmission of strains at the interface of rural, urban, and more natural environments.

## Data Availability Statement

The raw data supporting the conclusions of this article will be made available by the authors, without undue reservation, to any qualified researcher.

## Ethics Statement

The animal study was reviewed and approved by Animal Care Committee at the University of Guelph.

## Author Contributions

SM led all aspects of laboratory and downstream analyses and drafted the manuscript. BH participated in laboratory and downstream analyses and assisted with manuscript preparation. KB participated in study design and fieldwork and assisted with manuscript preparation. VG contributed to study design and manuscript preparation. CJ conceived of the study, participated in its design and coordination, and assisted with manuscript preparation. ET participated in study design and coordination, contributed to downstream analyses, and manuscript preparation.

### Conflict of Interest

The authors declare that the research was conducted in the absence of any commercial or financial relationships that could be construed as a potential conflict of interest.
